# The association between long term intake of ultra-processed foods and recurrence of depressive symptoms in the Whitehall II cohort

**DOI:** 10.1192/j.eurpsy.2022.552

**Published:** 2022-09-01

**Authors:** H. Arshad, T. Akbaraly

**Affiliations:** 1 Universite Paris Saclay, Centre De Recherche En Épidémiologie Et Santé Des Populations, Umr 1018, Paris, France; 2 Université Paul-Valéry, Maison Des Sciences De L’homme Sud, Montpellier, France

**Keywords:** depressive symptoms, Prospective study, Ultra-processed foods, diet

## Abstract

**Introduction:**

High amounts of Ultra-Processed Foods (UPF) characterized Western type diet and have recently been associated with adverse cardio-metabolic outcomes. The extent to which UPF intakes affect Depressive Symptoms (DepS) in non-Mediterranean countries remains uninvestigated.

**Objectives:**

We aimed to study whether long-term intake of UPF over adult life 1) is associated with subsequent recurrence of DepS assessed over 13 years of follow-up and 2) contribute to explain the diet quality-DepS associations already established.

**Methods:**

Data came from the 4554 participants (mean age=61.0 (SD=5.9) years; 74% men) from the Whitehall II Study who underwent repeated dietary intake assessment (food frequency questionnaire in 1991-1993, 1997-1999 and 2002-2004), and follow-up for recurrence of DepS (CES-D ≥ 16 or use of antidepressants) over 13 years (2002-2004 and 2015-2016). The NOVA classification was used to estimate UPF intakes.

**Results:**

Over 13 years of follow-up, 12.9% of participants reported having recurrence of DepS. Results of logistic regression models adjusted for potential confounders showed that high amounts of UPF intakes (top quintile versus the four last ones) increased the odds of recurrent DepS by 30 % (95%CI 1.05 - 1.61). Additional analyses suggested that UPF intakes did not attenuate much the overall diet quality–DepS association previously reported.

**Conclusions:**

Our study showed that long term exposure to high UPF intakes increased odds of subsequent recurrent DepS. This association was independent of overall diet quality. Further research is needed to understand the underlying mechanisms between food processing and depression physiopathology.

**Disclosure:**

No significant relationships.

